# Total Hip Arthroplasty When Utilised in Young Adults Is a Cost-Effective Procedure: A 10-Year and Lifetime Cost-Utility Analysis

**DOI:** 10.7759/cureus.22651

**Published:** 2022-02-27

**Authors:** Paul H. C Stirling, Navnit S Makaram, Nick D Clement, Deborah Macdonald, Gavin J Macpherson

**Affiliations:** 1 Edinburgh Orthopaedics, Royal Infirmary of Edinburgh, Edinburgh, GBR

**Keywords:** young, cost utility, cost effectiveness, quality of life, total hip replacement

## Abstract

Purpose

The primary aim of this study was to determine the cost-effectiveness of total hip arthroplasty (THA) in patients aged 25 years and under by calculating the cost per quality-adjusted life year (QALY) gained at 10 years post-operatively, and over the course of a lifetime. Secondary aims were to describe the change in health-related quality of life (HRQoL), Oxford hip score (OHS), and satisfaction in these patients.

Methods

From 2000 to 2016, 33 patients undergoing THA aged 25 and under had pre-operative and one-year post-operative EuroQol five-dimensions (EQ-5D) scores and OHS recorded prospectively. Post-operative change in EQ-5D allowed calculation of a health-utility score, which, when combined with life expectancy, gave total QALYs gained.

Results

The mean age was 20 years (range 13.3-24.9), with 23 females (72.7%). Mean number of QALYs gained was 21.1 (95% CI 14.1-28.2). Total lifetime cost per patient was £14641, giving a mean cost per QALY of £4183 at 10 years post-operatively, and £694 over the total remaining lifetime. Discounting total QALYs gained at a rate of 3.5% and 5% per remaining year of life expectancy increased the mean cost per QALY to £1652 and £2187, respectively. Mean pre- and post-operative EQ-5D index were 0.27 (SD 0.27) and 0.63 (SD 0.29), respectively (p=0.0001). Mean pre-operative and post-operative OHS was 37.5 (SD 7.9) and 19.7 (SD 6.94), respectively (p<0.00001).

Conclusion

THA remains a cost-effective intervention for patients aged 25 years and under. It is also associated with significant improvement in HRQoL, OHS, and high levels of patient satisfaction in this unique patient group.

## Introduction

The number of patients undergoing total hip arthroplasty (THA) is increasing [1] and the average age of these patients is decreasing. Although patients aged under 30 still only account for 1% of all THAs performed [2], the projected increased demand for THA in younger patients is likely to lead to increased requirements for revision THA [3], due to longer projected life expectancy in this age group. Numerous studies have investigated survivorship of THA in younger patients, with patients aged 40 or 50 years and younger typically investigated. Surgeons may cite young age at primary THA as a risk factor for future revision, and the survivorship and functional outcomes of THA in “super-young” patients aged 25 years and under is not well understood. This age range represents a patient cohort with unique functional requirements when compared to older patients, which may influence patient expectations and satisfaction [1]. In addition, many patients requiring THA under the age of 25 years will have undergone previous hip-preserving surgery, which may increase the complexity of the procedure [4] or the complication and revision rate [5].

Achieving pain relief and improving health-related quality of life (HRQoL) are the fundamental objectives of primary THA, and have been shown to influence patient satisfaction [6]. However, the use of patient-reported functional outcome measures (PROMs) as a surrogate for patient satisfaction and cost-utility following THA has been called into question [7]. As a result, the vast majority of studies of young patients undergoing THA focus on survivorship rather than improvement in HRQoL and functional outcomes after primary THA [8].

The cost-effectiveness of an intervention can alternatively be determined by defining the cost per quality-adjusted life year (QALY) gained after the procedure. This method is used by the National Institute for Health and Clinical Excellence (formerly NICE) as the principal measure of health benefit and cost-utility following a procedure [9], and has previously been used to quantify the cost per QALY following THA in a standard adult population [10].

The primary aim of this study is to investigate the cost-effectiveness of primary THA in patients aged 25 years and under by determining the cost per QALY gained at 10 years post-operatively and over the course of the patient’s lifetime. The secondary aims are to characterise the HRQoL and early hip-specific functional outcomes of these patients. 

## Materials and methods

Ethical approval was obtained from the regional ethics committee (Research Ethics Committee, *BLINDED*) for collection, analysis, and publication of the anonymised data collected for this study. The setting for this study was a large teaching hospital, which serves as the regional referral unit for complex hip arthroplasty cases, and serves an urban population of 810,000 [11]. This was a prospective study over a 16-year period (2000-2016). During the study all patients undergoing primary THA had demographic and outcome data recorded prospectively at the pre-operative assessment clinic. They were subsequently contacted one year post-operatively via postal questionnaire. The inclusion criterion for this study was a primary THA utilising a cemented femoral stem in patients aged 25 years and under with available outcome data recorded pre-operatively and one year post-operatively. Exclusion criteria were dislocation in the first year (n=0), THA utilising an uncemented femoral stem (n=2), and endoprosthetic replacement for malignancy (n=1). During the study period a consultant surgeon performed or scrub-supervised all included THAs. A total of 10 different consultant surgeons performed the procedures. Thirty-two patients received a cemented Exeter V40 stem (Stryker, Kalamazoo, Michigan) and one patient received an Olympia stem (Zimmer Biomet, Warsaw, Indiana). Thirty patients received a cemented Exeter Contemporary acetabular component (Stryker, Kalamazoo, Michigan), and three patients underwent hybrid THA with an uncemented acetabular component. The modified Hardinge or posterior approaches were used for all cases. All patients received three peri-operative doses of prophylactic antibiotics (cefuroxime) and six weeks of post-operative pharmacological deep venous thrombosis prophylaxis. A standardised rehabilitation protocol was used for all patients, with active mobilisation and full weight-bearing on the first day post-operatively. Patients were then reviewed at six weeks, six months, and 12 months post-operatively.

Post-operative outcomes

The EuroQol five-dimensions (EQ-5D) [12] general health questionnaire and the Oxford hip score (OHS) [13] were recorded pre-operatively and at one year post-operatively. The EuroQol (EQ) general health questionnaire evaluates five domains, which include mobility, self-care, usual activities, pain/discomfort, and anxiety/depression. This generates a unique index based on patient responses which is standardised for population and ethnicity. The index is converted to a score from -1.0 (a state worse than death) to 1.0, which represents a perfect health state. The OHS consists of 12 questions assessed on a Likert scale with values from 0 to 5. A summative score is then calculated where 12 is the best possible score (least symptomatic) and 60 is the worst possible score (most symptomatic). Patient satisfaction was assessed by asking the question “How satisfied are you with your operated hip?” one year after surgery. The response was recorded using a four-point Likert scale: very satisfied, satisfied, uncertain, and unsatisfied. Patients who recorded very satisfied or satisfied were classified as satisfied.

Cost per QALY analysis

To determine the cost-effectiveness of THA in young patients, decision tree analysis methodology was undertaken with individual time-dependency and revision risk calculated individually for each patient based on their life-expectancy. The final health state was full health with intact implant, revision arthroplasty, or death. Utilising the change in EQ-5D index allowed calculation of the cost-effectiveness of THA in young patients by allowing a cost per QALY gained to be calculated. This methodology has previously been described and applied to THA and total knee arthroplasty in adult patients [10]. The health state index, derived from the improvement in EQ-5D, is multiplied by the number of years spent in that state to calculate the number of QALYs gained or lost. The number of years spent in that state is determined by consulting national life tables [14], which vary based on the patient age and gender, to predict the remaining life expectancy. This analysis assumes that health status remains fixed and that there no requirement for further intervention within one year [15]. Total QALYs gained were then discounted at a rate of 3.5% and 5% per year of life expectancy, to account for a projected diminishing gain over time [16]. The cost of THA was defined as £7137 according to the Scottish National Tariff [17]. The tariff for aseptic revision was £9048 and septic revision was £14 441 [17]. These costs account for bed days and other associated health-care costs. The likelihood of revision THA for each patient was assigned as 0.5% up to 15 years (7.5% revision rate at 10 years) and 1.5% per year beyond that [10], which were uniformly applied for all patients regardless of their age under 25. The predicted proportion of septic revisions for THA was set at 15% in line with previous research [10,18]. The total cost of treatment for each patient could, therefore, be predicted, and this was divided by the number of patients to determine the mean cost per procedure per patient. Dividing this by the number of QALYs gained results in the overall cost per QALY, which is adjusted to give cost per QALY at 10 years post-operatively and over the course of a lifetime.

Statistical analysis

Statistical analysis was performed using Statistical Package for Social Sciences version 17.0 (SPSS Inc., Chicago, IL, USA). Data were checked for normality using the Shapiro-Wilk test. Parametric tests were used for normal data, which is reported as mean and standard deviation (SD). Non-parametric data are reported as median and interquartile range. Pre- and post-operative outcomes and EQ-5D were compared using a paired t-test. Correlation between OHS and EQ-5D index was assessed using Spearman’s correlation. A p-value <0.05 was considered statistically significant.

## Results

Patient cohort

There were 33 THAs performed during the study period that met the inclusion criteria. The mean age was 20 (range 13.3-24.9) years, and there were 23 females (72.7%). The most prevalent comorbidities were juvenile idiopathic osteoarthritis (27%), followed by avascular necrosis and congenital hip dysplasia (both 24%).

Primary outcome

Mean pre- and post-operative EQ-5D index were 0.27 (SD 0.27) and 0.63 (SD 0.29), respectively (p=0.0001) (Table 1).

**Table 1:** Patient-reported outcomes at pre-operative follow-up, post-operative follow-up, and change in score N/A: not available; SD: standard deviation; 95% CI: 95% confidence interval; EQ-5D: EuroQoL five-dimensions score

Outcome	Pre-operative (n=33)	Post-operative 1-year (n=33)	p-Value
OHS (mean (SD))	37.5 (7.9)	19.7 (6.94)	<0.0001
Change in OHS (mean (95% CI))	N/A	17.8 (14.6-21.0)	N/A
EQ-5D (mean (SD))	0.27 (0.27)	0.63 (0.29)	0.0001
Change in EQ-5D (mean (95% CI))	N/A	0.35 (0.234-0.466)	N/A

Mean improvement in EQ-5D was 0.35 (SD 0.34; range -0.43 to 1.02). Mean life expectancy was 60.7 years (SD 4.2 years). Mean number of QALYs gained was 21.1 (95% confidence interval 14.1-28.2; range -28.6 to 62.7) (Table 2). Total lifetime cost per patient was £14641, giving a mean cost per QALY of £4183 at 10 years post-operatively. Mean cost per QALY over the patients predicted remaining lifetime was £694. Discounting total QALYs gained at a rate of 3.5% and 5% per remaining year of life expectancy increased the mean cost per QALY to £1652 and £2187, respectively, over the remaining life expectancy (Table 2).

**Table 2:** Lifetime, five-year, and 10-year cost-utility analysis of total hip arthroplasty QALY: quality-adjusted life year; EQ-5D: EuroQoL five-dimensions score; SD: standard deviation; 95% CI: 95% confidence interval

Health-related quality of life scoring system and consequential costs	Associated outcome
Preoperative EQ-5D (mean, SD)	0.27 (0.27)
Post-operative EQ-5D (mean, SD)	0.63 (0.29)
EQ-5D improvement (95% CI)	0.35 (0.234-0.466)
Mean (SD) life expectancy (years)	60.7 (4.2)
Mean QALYs gained in years (95% CI) range	21.2 (14-28.2) (-28.6 to 62.7)
Approximate mean procedural cost per patient	£14641
Lifetime cost per QALY	£694
Lifetime cost per QALY (discounted 3.5%)	£1652
Lifetime cost per QALY (discounted 5%)	£2187
Cost per QALY at 5 years	£8366
Cost per QALY at 5 years (discounted 3.5%)	£8973
Cost per QALY at 5 years (discounted 5%)	£9246
Cost per QALY at 10 years	£4183
Cost per QALY at 10 years (discounted 3.5%)	£4885
Cost per QALY at 10 years (discounted 5%)	£5212

Secondary outcomes

Mean pre-operative and post-operative OHS were 37.5 (SD 7.9) and 19.7 (SD 6.94), respectively (p<0.0001) (Table 1). Mean post-operative improvement in OHS was 17.8 (SD 9.3). There was no significant correlation between age at the time of surgery and change in EQ-5D and OHS, or pre- and post-operative EQ-5D and OHS. Improvement in OHS correlated significantly with improvement in EQ-5D index (r=0.3991; p<0.05). Thirty patients (90.9%) were satisfied following their hip replacement.

Complications

Three patients (9.1%) required further surgical intervention at a mean 8 years follow-up (range 2.6-19.5 years). Two patients required revision THA, both for aseptic failure of the acetabular component. One patient sustained a Vancouver type C periprosthetic femoral fracture. This required open reduction and internal fixation (ORIF), with three subsequent ORIF procedures required for hardware breakage.

## Discussion

Having factored in lifetime revision risk, and conservatively discounting QALYs gained at a rate of 5% per year of remaining life expectancy, the cost per QALY still remains within the threshold of £20000 to £30000 above which the National Institute for Health and Clinical Excellence is reluctant to fund treatment [19]. Compared with a standard population of adult patients, patients aged under 25 who underwent THA reported a cheaper unadjusted cost per QALY at 10 years (£4183 per QALY) and over the course of a lifetime (£694) [10]. Though the cost at outset is cheaper in the first 10 years following THA, the overall lifetime cost per QALY becomes more expensive once the QALYs gained are discounted at a rate of 3.5% and 5%. This may be partially explained by considering the lower age of this patient cohort. In the previous cost-utility analysis of THA published by Jenkins et al., the mean age was 66.1 years, and mean life expectancy was 18.7 years [10]. By comparison, the mean age in our study was 20 years and mean life expectancy was 60.7 years, which may explain the higher lifetime cost per QALY observed in our study due to cumulative reduction in HRQoL year on year. In reality, we hypothesise that HRQoL would not deteriorate to this level as patients would be more likely to undergo revision surgery than deteriorate for the remainder of their life. We account for this with a probabilistic calculation of lifetime risk of revision, but due to lack of evidence regarding HRQoL following revision THA, we were unable to accurately predict the impact of this on HRQoL. It has been suggested previously that the cost per QALY for THA would depend on case-mix variables with specific emphasis on age, which may alter the number of QALYs gained and, therefore, the cost-effectiveness of the procedure [20]. Our results demonstrate that THA remains a cost-effective procedure even in very young patients utilising QALY methodology. 

It has been reported that outcomes following THA are poorer in younger patients [21], yet the vast majority of research justifying this conclusion focuses on survivorship rather than PROMs. Patients aged 40 [22] or 50 [23-25] years and younger are often examined in the same cohort, making it difficult to accurately describe the functional outcomes in patients aged under 25. A further finding of this study is a significant improvement in OHS in this group of patients. Average OHS improvement was 17.8 points and no patients reported a deterioration in OHS following THA. This observed improvement is greater than the previously reported minimum clinically important difference for the OHS which has been reported as 11 points [26], suggesting that these results were not only statistically, but also clinically significant. In addition, the mean post-operative OHS, which would be classified as good joint function according to the original description of the OHS [9], is comparable with previously reported OHS following THA in older patients [27], and is higher than previously reported threshold values for satisfaction following THA [28]. This suggests that very young patients undergoing THA using cemented stems can expect good functional improvement following their surgery.

THA in this group of patients has historically been reserved as a treatment modality of last resort for prolonged debilitating arthritis interfering with quality of life. The first studies investigating this suggested reasonable functional and psychological improvements could be achieved in patients with juvenile idiopathic or rheumatoid arthritis using cemented Charnley stems [29,30]. These early studies are of limited clinical value today given the significant developments in prosthesis design and PROM analysis that have subsequently occurred.

The main limitation of this study is the use of national tariffs to determine the procedural cost. This methodology is in line with previous studies defining cost per QALY for THA [10], but may not reflect specific local costs, and in particular the costs of revision surgery, which may be particularly relevant in this patient cohort. A further limitation is the lack of data relating to HRQoL following revision THA. This study has used a simple probabilistic approach to determine the total lifetime risk of revision in this patient cohort, in line with a previous health-economic analysis of THA [10]. This may not be applicable to this specific patient cohort; however, as given the long remaining life expectancy of the cohort, they would be expected to outlive their implant, and therefore the revision rate for this cohort may be higher than that of older adults. It was, therefore, not possible to quantify the impact of a possible reduction in HRQoL associated with revision THA in this cohort. The small sample size is a further limitation of this study, which may increase the risk of a type 2 error.

## Conclusions

This study demonstrates that THA is a cost-effective intervention for patients aged 25 years and under. THA also confers a clinical and statistically significant improvement in OHS and HRQoL in this unique patient cohort. Further multi-centre prospective studies incorporating larger sample sizes should be conducted to further corroborate this study's findings.

This study has shown that THA using contemporary cemented polished taper-slip stems is a cost-effective procedure, which leads to excellent functional outcomes in very young patients, aged 25 years and under.
